# From Carbon to Plastics: Partitioning of Biogeochemical and Anthropogenic Particles in Penguin Guano

**DOI:** 10.1021/acs.est.5c17586

**Published:** 2026-06-12

**Authors:** Erica Sparaventi, Emily Rowlands, Federico Giglio, Araceli Rodríguez-Romero, Antonio Tovar-Sánchez, Clara Manno

**Affiliations:** † 41820 British Antarctic Survey, High Cross, Madingley Road, Cambridge CB3 0ET, U.K.; ‡ Department of Genetic Toxicology and Cancer Biology, National Institute of Biology, Večna pot 121, Ljubljana 1000, Slovenia; § Department of Ecology and Coastal Management, Institute of Marine Sciences of Andalusia, ICMAN (CSIC), Campus Río San Pedro, Puerto Real, Cádiz 11510, Spain; ∥ CNR-ISP—National Research Council of Italy—Institute of Polar Sciences, Bologna 40129, Italy

**Keywords:** carbon, microplastics, penguins, guano, Antarctic, biogeochemical cycle

## Abstract

Penguins are emblematic inhabitants of the Antarctic continent and play a significant role in the biogeochemical cycles of the Southern Ocean by transferring essential nutrients to marine ecosystems via guano production. Despite their ecological importance, the contribution of penguin guano to carbon cycling remains largely unexplored. Microplastics (MPs) add complexity to these dynamics. MPs in fecal material can limit carbon export by reducing sinking rates and increasing remineralization. We examined guano from two penguin species, the Chinstrap (*P. antarcticus*) (*n* = 25) and Gentoo (*P. papua*) (*n* = 7), from the South Shetland Islands. We quantified biogeochemical particulate components (carbon, nitrogen, and biogenic silica) and characterized MP polymeric composition. Both species showed similar values for natural particulate fractions. Microplastics were detected in 91% of samples, dominated by small particles (25–50 μm, 46%). Chinstrap guano contained the highest amount of MPs. Polypropylene was the predominant polymer (34% in Chinstrap, 75% in Gentoo), followed by polyethylene (37% in Chinstrap, not found in Gentoo). This study provides the first survey of the smallest MP fraction (down to 25 μm) in penguin guano, offering new insight into how a shift in the partitioning from natural to MP particles, previously overlooked, may influence guano-mediated carbon pathways.

## Graphical abstract

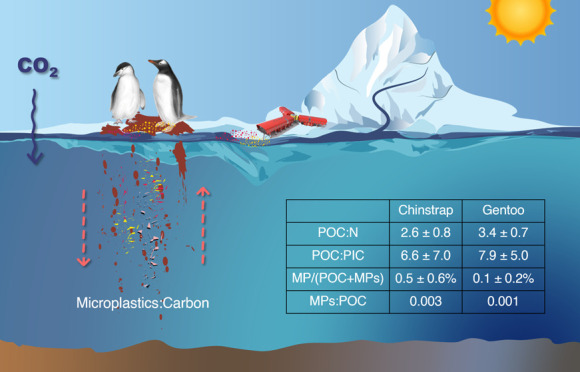

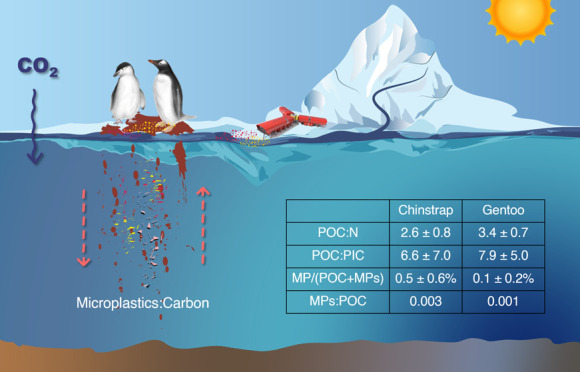

## 1 Introduction

Penguins represent 90% of seabirds’ biomass in the Southern Ocean (SO).^1^ Of the 18 existing species, five inhabit the Antarctic continent, including three from the genus *Pygoscelis*, the Adélie (*P. adeliae*), Chinstrap (*P. antarcticus*), and Gentoo (*P. papua*). These are considered the sentinel indicators of marine ecosystem health due to their large populations (estimated at 13.5 million individuals^2^) and their circumpolar distributions. Spending about 75% of their life at sea and occupying a middle position in the Antarctic food web, they predominantly feed on Antarctic krill (*Euphausia superba*, hereafter krill) and are effective indicators of ocean health.^3,4^ Penguin guano is the main source of macronutrients (e.g., nitrogen and phosphorus) and micronutrients (e.g., trace metals) on the Antarctic land.^5^ Furthermore, recent studies highlighted the important role of penguin guano in SO biogeochemical cycles. The release of dissolved trace metals (e.g., iron, zinc, and copper) mediated by guano enriches the upper layers of the iron-limited SO.^6,7^ This input enhances phytoplankton communities’ growth, stimulating primary production.^7–9^

The SO plays a key role in global climate system regulation, by coupling physical (e.g., circulation dynamics, upwelling of nutrient-rich waters, and the formation of deep and intermediated water masses that transport carbon to the ocean floor) and biological processes that drive both local carbon fixation and long-term carbon sequestration.^10–12^ In particular, previous studies showed that fecal material produced by organisms at a low trophic level (i.e., zooplankton) represents a fundamental route in the SO for carbon to reach the deep ocean, supporting oceanic carbon sequestration.^13–16^ However, the potential contribution of feces produced by upper pelagic predators in the Antarctic ecosystem, including penguin guano, in mediating the downward flux of carbon to the oceanic depths remains almost unexplored. Understanding this contribution could provide valuable insights into the complex mechanisms that regulate carbon export dynamics in the SO.

Microplastic (MP, size between 0.1 μm and 5 mm) pollution further complicates these dynamics. The presence of MPs within fecal pellets can attenuate sinking rates, thereby enhancing remineralization and limiting vertical carbon export.^17–19^ Recent evidence reveals that exposure to polystyrene nanoparticles (NPs, size <1 μm) accelerates krill fecal pellet degradation, potentially reducing carbon sequestration by up to 27% (5.5 Mt. C) per productive season.^20,21^ Microplastics have been detected in penguin guano from different species (Gentoo, Adélie, Chinstrap, and King penguins) and locations (South Georgia, South Orkney Islands, and across the Antarctic Peninsula).^22–24^ Dietary intake is identified as the primary pathway for MPs, through accidental direct ingestion or indirectly via krill consumption.^24,25^ MPs in the SO may originate from local sources, including ship traffic and Antarctic research facilities,^26^ as well as from distant lower-latitude seas,^27^ with meridional transport across the Antarctic Circumpolar current. While MP concentrations may be minimal when averaged across the SO,^28^ they can be relatively high in close proximity to local emission sources, particularly in coastal areas nearby research facilities and shipping routes, where they may pose a localized ecological risk.^26,29–31^

Here, we investigated the particulate composition of guano from two key Antarctic penguin species, Chinstrap and Gentoo, at Deception and Livingston Islands (South Shetland Islands, off the Antarctic Peninsula). We provide the first integrated quantification of both the biogeochemical (organic carbon (POC), inorganic carbon (PIC), total nitrogen (TN), and biogenic silica (BSi)) and anthropogenic (MP) particulate composition. We identified the MP polymer type down to a size of 25 μm, an unprecedented resolution for such samples in penguin guano, despite most of the environmental available MPs in Antarctica having a size smaller than 50 μm.^32,33^ Finally, we examined the guano carbon–MPs fraction interaction and the potential implications for the carbon cycle.

## 2 Materials and Methods

### 2.1 Penguin Guano Samples Collection

A total of 32 fresh penguin guano samples were collected during two Antarctic campaigns in the South Shetland Islands (SSI). In the austral summers of 2020/2021 and 2021/2022, samples were collected from Chinstrap penguin breeding colonies at Vapour Col (62°59′S, 60°44′W) (20,000 breeding pairs,^34^) and Baily Head (62°58′S, 60°30′W) (50,000 breeding pairs,^35^) on Deception Island (*n* = 25) (Figure 1). In the austral summer of 2021/2022, samples were collected from Gentoo penguin breeding colonies at Hannah Point (62°39′S, 60°36′W), Sally Rock (62°42′S, 60°25′W), and Argentina Cove (62°40′S 60°24′W) on Livingston Island (*n* = 7) (Figure 1). Single colony census is not available for Gentoo penguins since they are distributed across multiple smaller colonies, but total abundance is estimated to be on the order of several thousand breeding pairs (10^3^–10^4^), based on site-specific monitoring data on Livingston Island. Wet guano was carefully collected by hand or with a plastic (polystyrene, PS) spoon from snow, ice, or rocks, avoiding collecting ground remains, placed in a conical centrifuge tube or in a zip-lock bag, and frozen at −20 °C prior to processing in the laboratory. Permissions to work and collect guano samples in the study area were granted by the Spanish Polar Committee.

**Figure 1:**
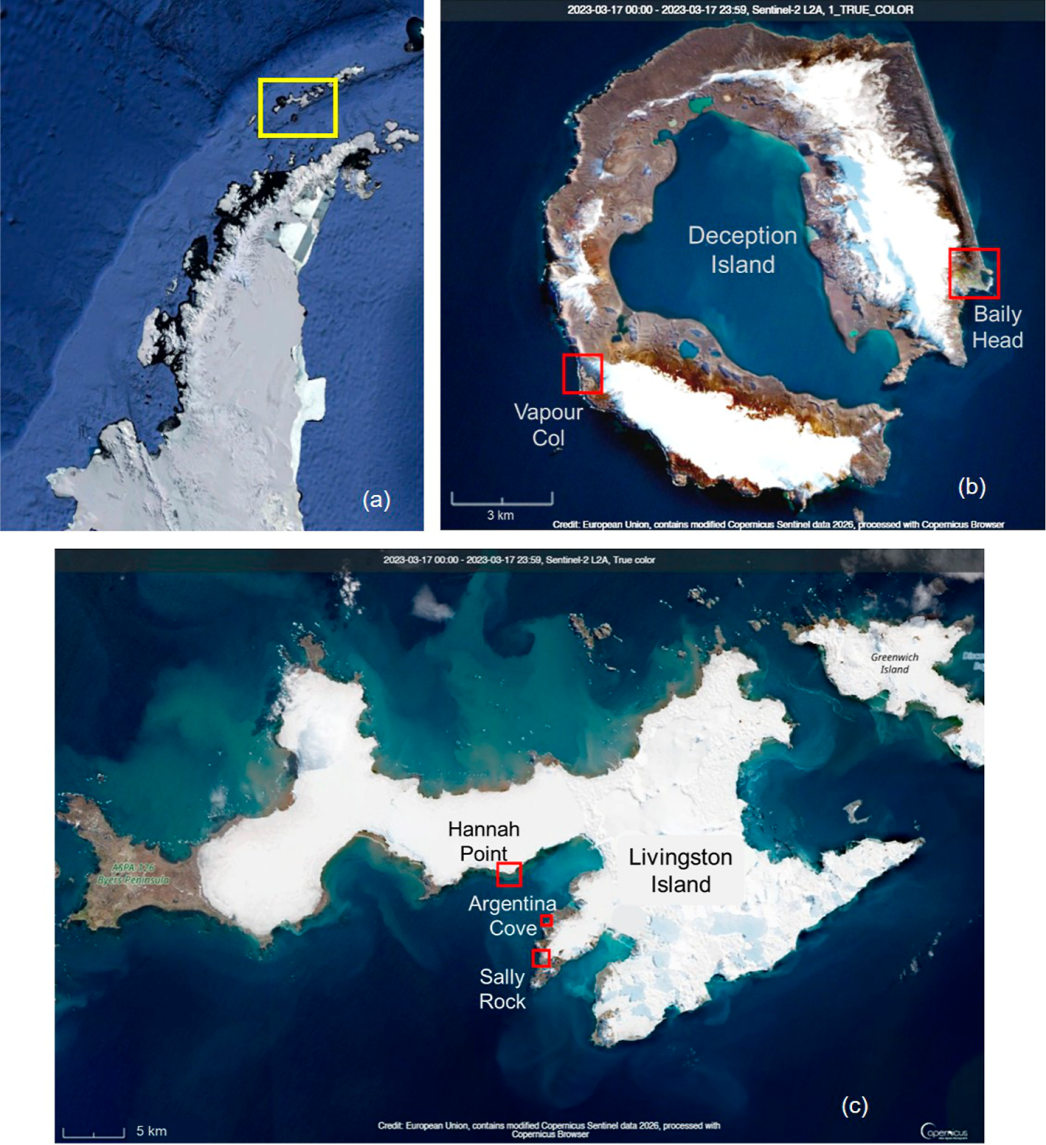
Maps showing sample collection areas. (a) The Antarctic Peninsula with the South Shetland Islands, the yellow square indicates the two areas considered for the sampling; (b) Sentinel 2A scene of Deception Island on March 17, 2023, the red squares indicate the colonies where Chinstrap penguin guano samples were collected during the austral summers 2020/2021 and 2021/2022; and (c) Sentinel 2A scene of Livingston Island on March 17, 2023, the red squares indicate the colonies where Gentoo penguin guano samples were collected during the austral summer 2021/2022. The size of each square is proportional to the population of penguins in each colony.

### 2.2 Quality Assurance/Quality Control (QA/QC)

To avoid potential contamination, all handling steps were performed under controlled clean conditions. Guano samples were collected with PS spoons and stored using falcon tubes and zip-lock bags; however, all storage materials were new and the spoon was cleaned after each use. Samples were handled to minimize external contamination prior to frozen condition storage, with exposure kept to a minimum. Once in the lab, for the microplastic particles extraction procedures, all reagents were first filtered through a 2 μm polycarbonate filter. All glass and beakers were cleaned with 95% ethanol and ultrapure water before use. Furthermore, all the steps included in the protocol were performed in a laminar flow hood in a restricted access laboratory, and only cotton lab coats and nitrile gloves were used. To check for contamination, three types of blanks were performed: (i) procedural blanks (3 replicates); (ii) lab contamination air blanks (2 replicates); and (iii) storage blanks, for zip-lock bags (2 replicates) and falcon tubes (2 replicates). In particular, a total of 3 procedural blanks were performed for the entire analysis and processed with the same protocol as that used for the samples but using only filtered Milli-Q. In addition, lab contamination air blanks used polycarbonate filters (47 mm diameter and 0.2 μm pore size) placed in glass Petri dishes on the laboratory bench and simulated all the steps and procedures that have been carried out. Furthermore, storage blanks, consisting of 2 empty zip-lock bags and 2 falcon tubes stored for over a year, were analyzed to check potential contamination during storage. No field blanks were taken to evaluate background contamination since guano sampling was not initially designed for investigating plastic pollution.^23^

### 2.3 Biogeochemical Analysis

Once in the lab, the 32 guano samples were freeze-dried (LABCONCO, model 798,030). Subsamples of dried guano (3.5 mg) were used for analyses of particulate biogeochemical matter. Total particulate carbon (TC), particulate organic carbon (POC), and total nitrogen (TN) were measured in triplicate by combustion in an elemental analyzer (CHN, Exeter Analytical Inc. CE440 elemental analyzer, accuracy ±0.15%).^36^ For POC determination, samples were pretreated with 1 N hydrochloric acid (HCl, analytical grade, ≥37%, then diluted, Sigma-Aldrich). Particulate inorganic carbon (PIC) was obtained by determining the difference between TC and POC. Three blank filters (GF/F filters, 25 mm, 0.45 μm pore size) were interspersed regularly between samples (run every 25 samples) to measure and correct for drift. The amount of particulate biogenic silica (BSi) in guano was quantified by colorimetric analysis (Jasco UVIDEC accuracy ±1%). For the silicate extraction, approximately 10–20 mg of the guano sample was suspended in 50 mL of 0.5 M sodium hydroxide (NaOH, pellets, Carlo Erba, Milan, Italy) at 85 °C for 5 h. Following a progressive dissolution method, an aliquot of each sample was taken for analysis every hour, and the relative silica data were extrapolated back to time zero to correct for the silica originating from coexisting clay minerals.^37^ The dissolved silicate was then reacted with ammonium molybdate tetrahydrate (99+%, Thermo Scientific). To shift the solution toward blue (measured at 810 nm), a reducing solution was added, composed of metol sulfite (sodium sulfite anhydrous, Thermo Scientific), 4-methylaminophenol sulfate (99% extra pure, Thermo Scientific), oxalic acid dihydrate (VWR), and sulfuric acid (H_2_SO_4_, diluted 50%, 96% purity, Panreac AppliChem).

### 2.4 Microplastics Extraction

We adapted and further improved a methodology used for the extraction and identification of MPs in penguin guano^24^ to enable the analyses of smaller MPs (>25 μm) (Figure 2). This utilized alkaline digestion, which has been proposed as an effective and cheaper option to enzymatic digestion as it is also less damaging for plastic particles or fibers.^38,39^ An initial optimization step was performed to determine the appropriate sample mass required to ensure complete digestion of the guano matrix. Different sample weights were tested, after which a standardized mass of approximately 1.5 g, was selected and used for subsequent analyses. The 32 freeze-dried samples were weighed (1–3 g), transferred into clean glass beakers with a 10% potassium hydroxide (KOH) solution, and placed on a shaker for 72 h at room temperature. The volume of KOH added was 3 times the volume of the biological material. After 72 h, the floating phase was separated from the settled part, by filtration onto separate 10 μm metal meshes using a glass filtration setup (45 mm) with a vacuum pump. Due to the large amount of organic matter (mainly due to the presence of a large volume of krill cuticles), the filters from both the floating and settled parts were placed in clean beakers, and a further 10% hydrogen peroxide (H_2_O_2_) solution was added for 24 h. The volume of the solution depended on the amount of the sample, up to a maximum of 100 mL. The digested floating phase filters were then vacuum filtered on Anodiscs (Anopore Inorganic Membrane, 0.2 μm, 25 mm, Whatman) to allow a full scan of the filter surface for the analysis of the smaller plastics (detection limit down to 11 μm) using a Focal Plane Array (FPA)-based imaging. The settled parts were vacuum filtered onto another 10 μm metal mesh. The meshes from the settled parts were systematically examined using a stereomicroscope LEICA M80 (Leica Microsystems GmbH) to identify potential MPs particles and/or fibers candidates, which were then hand-picked using forceps and placed onto the Anodiscs for the further analysis. For the selection of possible MPs, the criteria proposed by Hidalgo-Ruz et al.^40^ have been followed as guidelines (i.e., choosing fibers with equal thickness and homogeneous colors).

**Figure 2:**
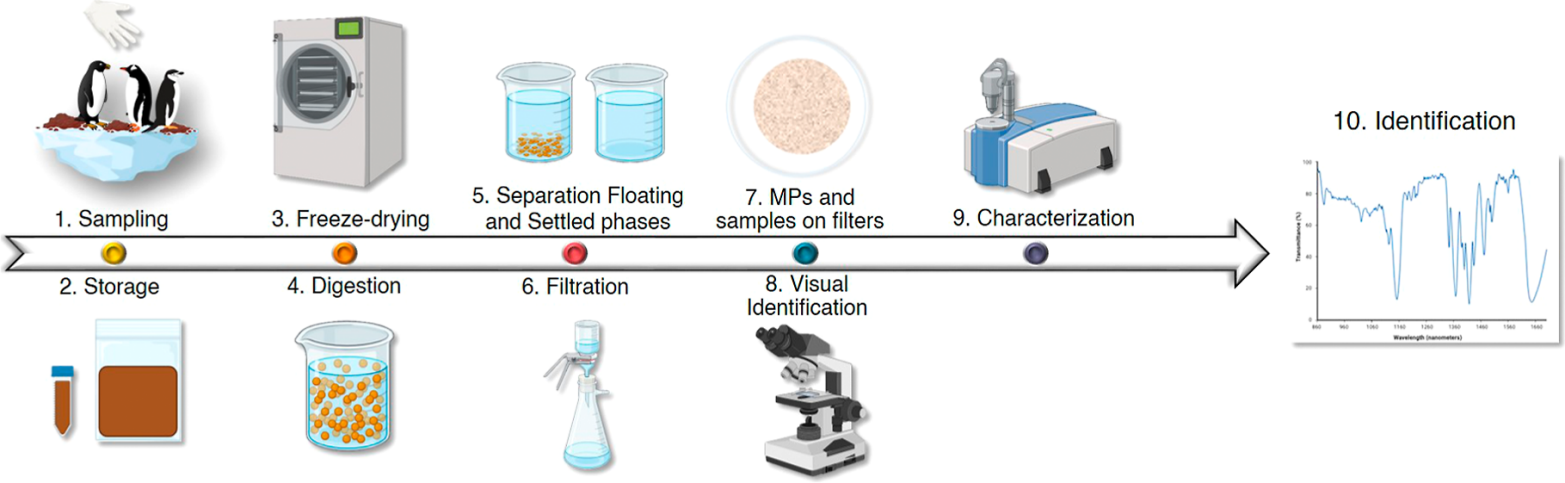
Schematic illustrations showing the steps involved in the methodology employed for the extraction, characterization (with a μ-FTIR spectrometer), and identifications of MPs in penguin guano.

### 2.5 Microplastics Characterization

A total of 11 digested floating phase Anodiscs were randomly selected from the 32 digested samples, five of Chinstrap penguin guano and six of Gentoo penguin samples, and analyzed for the identification of MP particles using FPA-based imaging. To identify the chemical composition of the particles/fibers, the Anodisc filters were analyzed using the Agilent 670 micro-Fourier Transform Infrared Spectroscopy system (μ-FTIR, Agilent Technologies, Santa Clara, CA, USA). The spectrometer was coupled to an Agilent 620 microscope with an automated XYZ-stage and a 128 × 128 focal plane array (FPA) detector, cooled with liquid nitrogen, in the transmission mode. FTIR spectral analysis was conducted in the spectral range of 3600–1250 cm^–1^ (spectral resolution: 8 cm^–1^). Each filter was fully analyzed by sequentially scanning one-half at a time to ensure accurate FTIR measurement. Before each sample scan, a background scan was collected on a clean Anodisc in the same spectral range. Spectra were further analyzed via Purency Microplastics Finder (MPF) (Purency GmbH, Austria) running a machine learning model (by Hufnagl et al.),^41^ which supports the identification of 21 polymers (Supporting Information, Table S1). This post hoc spectral analysis in Purency used a relevance tolerance limit of 0.6, and spectral matches were filtered to match the lower size threshold of the analytical technique of 11 μm.

### 2.6 Microplastic Mass Calculations

The mass of MP particles was determined following the method presented in Rowlands et al.,^42^ where the calculation referred to fragments. For fragments, volume was calculated as a cross-sectional area multiplied by thickness. Area measurements derived from the Purency software, which account for concave regions of the 2D shape, were multiplied by fragment thickness (*T*) after adjusting thickness to account for potential (unobserved) concavity across the third dimension. The thickness was estimated from length and width measurements by assuming equality of the width/length and thickness/width ratios. The volume calculation was therefore *V* = *P* × *T* × area. Fragment mass was then calculated assuming the rough volumes of particles and by multiplying volume by the polymer density (Supporting Information, Table S2).

### 2.7 Statistical Analysis

Statistical differences in the biogeochemical fraction (POC, PIC, TN, BSi, POC/N, and POC/PIC) and the MP polymers (type, size, and the percentage of MPs relative to the total particulate MPs plus POC) between the two studied species have been analyzed using R studio software version 4.4.3 (2025-02-28 ucrt). Normality was tested using Shapiro–Wilk tests; when data met normality assumption, significant differences among species were assessed by one-way ANOVA; otherwise, the nonparametric analysis of variance Kruskal–Wallis was applied. The significance level was set at *p* < 0.05.

## 3 Results

### 3.1 QA/QC Results

The three types of blanks performed showed negligible microplastic presence. The procedural blanks (performed along the entire analysis) contained an average of 2 items per filter, with an average size of 61 ± 45 μm. The lab contamination air blanks contained an average of 2.2 particles per filter (with 94% PA) with an average size of 41 ± 15 μm (Supporting Information, Table S3). Only two polyamide (PA) particles were identified in the storage blanks, with sizes 39.9 and 62.6 μm. No microplastic items were found in falcon tubes blanks. Therefore, the potential contamination during storage and laboratory processing was minimal. The three types of blanks were used for normalizing the number of MPs extracted and identified from guano samples. For the procedural and storage blanks data correction, we subtracted the total number of MPs observed from the total number of MPs in samples due to matching in polymer composition and size. For the air contamination blank data correction, we subtracted the mean MP value reported in blanks from the mean MP value of samples.^43^

### 3.2 Guano Biogeochemical Composition

The relative abundance of the biogeochemical particulate components was similar for both penguin species (Figure 3a). Overall POC was the dominant component of the total guano biomass, followed by TN and PIC. BSi values were under the detection limits for all the samples. The POC concentration, analyzed for each sample in triplicate, from Chinstrap (*n* = 75) and Gentoo (*n* = 21) guano showed no significant difference (*F*_1_,_94_ = 0.25, *p* > 0.05), with the respective averages of 25.4 ± 4.1% and 26.0 ± 4.9% of their dry mass (dw). Significant differences were found in PIC (H(1) = 6.05, *p* < 0.05) and TN (H(1) = 7.43, *p* < 0.05) levels between guano of the two species, being higher in Chinstrap than in Gentoo. Chinstrap PIC average was 5.9 ± 3.4% and that of Gentoo was 3.9 ± 2.8%; TN levels were 10.6 ± 3.3% for Chinstrap and 7.9 ± 1.5% for Gentoo. POC:N and POC:PIC ratios were also significantly different (*H*(1) = 11.79, *p* < 0.001 and *H*(1) = 4.55, *p* < 0.05, respectively) between guano from the two species (Figure 3b). The average value of POC:N in Chinstrap was 2.6 ± 0.8 and that for Gentoo was 3.4 ± 0.7; POC:PIC in Chinstrap was 6.6 ± 7.0 and that in Gentoo was 7.9 ± 5.0.

**Figure 3:**
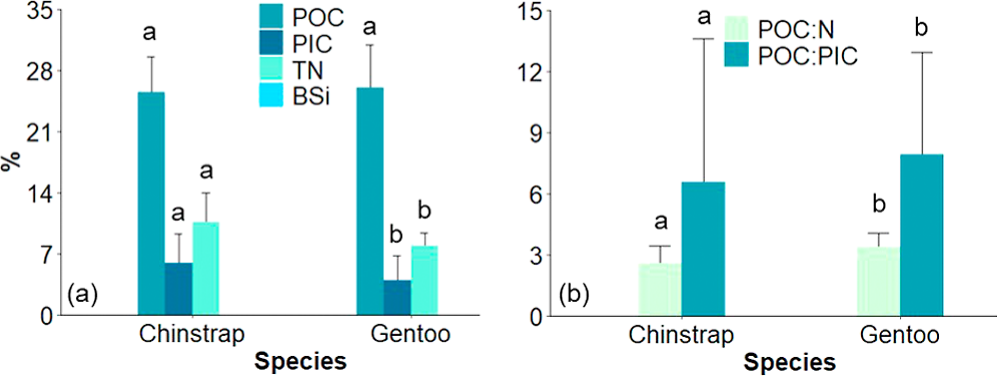
(a) Chemical particulate characterization expressed as % (of their dry mass) of POC, PIC, TN, and BSi and (b) expressed as POC:N and POC:PIC ratios of Chinstrap and Gentoo penguin guano collected off the Antarctic Peninsula during the austral summers 2020/2021 and 2021/2022. Different letters indicate significant differences (*p* < 0.05) between the two species.

### 3.3 Guano Microplastic Composition

Microplastic particles were found in 91% of guano samples (10 out of 11); the only MP-free sample was from Gentoo. In total, 101 MP particles were recovered, normalized on guano digested volume (MP/cm^3^). Chinstrap penguin showed a higher presence of MPs, with an average of 15 ± 14 MP/cm^3^ being found compared to an average of 4 ± 2 MP/cm^3^ across Gentoo samples (Supporting Information, Table S3), with significant differences found regarding the number of MP particles in guano between the two penguin species (*H*(1) = 11.851, *p* < 0.05). Seven different types of polymers were identified, with the highest frequency polymer being polypropylene (PP) found in 34% of Chinstrap and 75% of Gentoo samples. The second most frequent polymer was polyethylene (PE) with a frequency of 37% in Chinstrap, but it was not found in Gentoo. Polyamide (PA) frequency of occurrence was 18% in Chinstrap samples. Polystyrene (PS) was present in 8% in Chinstrap and 9% in Gentoo samples, while polyethylene terephthalate (PET) was found in 1% and 15%, respectively. Cellulose acetate (CA) with 1% and polyacrylonitrile (PAN) with 1% were the less common type of polymers, found only in Chinstrap guano samples. A total of 6 fibers were manually isolated. Chinstrap penguin samples showed a higher abundance, with 4 fibers (two PAN, one PA, and one PET), whereas in Gentoo samples were found 2 items (both PET fibers) (Figure 4).

**Figure 4:**
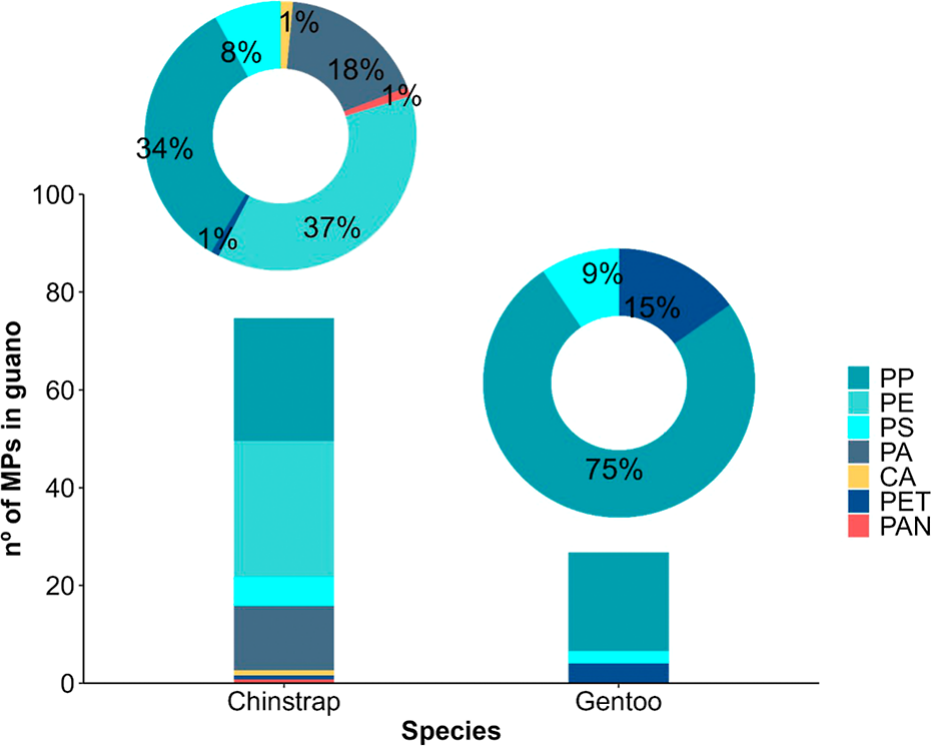
Total number and frequency of occurrence (%) of particles and fibers identified via μ-FTIR, normalized on guano digested volume (cm^3^) across the two penguin species, Chinstrap (*n* = 5) and Gentoo (*n* = 6), categorized by polymer type: polypropylene (PP), polyethylene (PE), polystyrene (PS), polyamide (PA), cellulose acetate (CA), polyethylene terephthalate (PET), and polyacrylonitrile (PAN).

The MPs class size range was from 25 μm to >5 mm, with the smallest (within 25–50 μm) being the most representative size class, with 68 MPs found (46% of frequency of occurrence), and they were more abundant in Chinstrap samples (41%) than in Gentoo (5%) (Figure 5). No significant differences were found between species and sizes in the Kruskall–Wallis test (*H*(1) = 0.019, *p* > 0.05). The identified fibers ranged in size from 1.3 to >5 mm (Figure 5).

**Figure 5:**
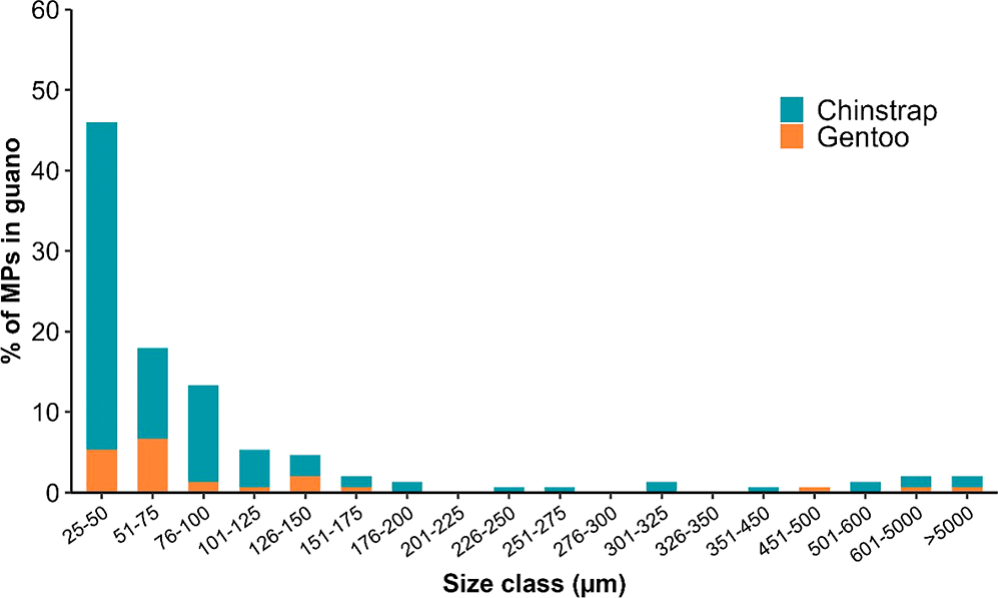
Frequency of occurrence (%) of MPs divided by size class (μm), found in penguin guano samples from Chinstrap and Gentoo penguins.

The mass of small MP particles calculated in each sample ranged from 0.05 to 11.6 μg, with an average of 4.0 ± 4.6 μg for Chinstrap samples and 1.5 ± 2.3 μg in Gentoo penguins (Figure 6). MP concentrations, expressed as μg mg^–1^ of guano dry weight (dw), varied between 0.0001 and 0.004 μg mg^–1^, with an average of 0.002 ± 0.002 μg mg^–1^ of guano (dw) in Chinstrap and 0.001 ± 0.001 μg mg^–1^ of guano (dw) in Gentoo (Supporting Information, Table S4). The percentage of MPs relative to the total particulate MPs plus POC was less than 2%, Chinstrap: 0.5 ± 0.6% and Gentoo: 0.1 ± 0.2%, with no significant difference between the two species (*H*(1) = 1.63, *p* > 0.05) and an average value of 0.3 ± 0.5% among the two species (Supporting Information, Table S4). This proportion increased to 3 ± 8% when plastic fibers >5 mm were also included.

**Figure 6:**
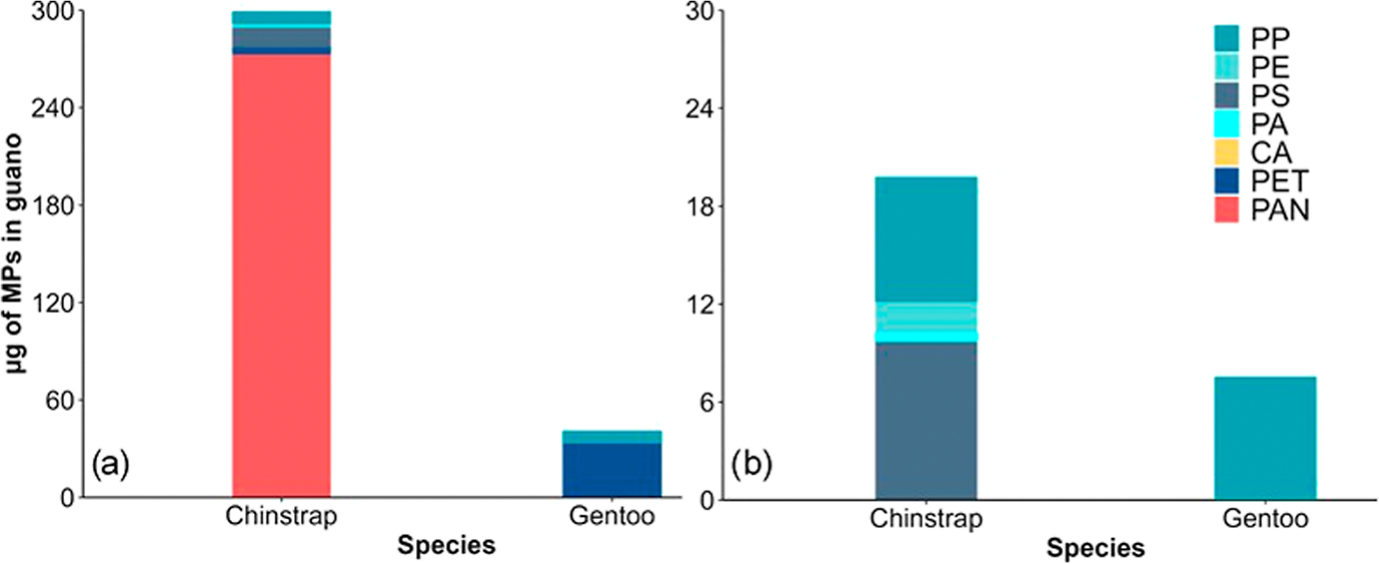
(a) Total weight (μg) of microplastic particles and fibers and (b) only small MP weight in guano digested samples across the two penguin species, Chinstrap (*n* = 5) and Gentoo (*n* = 6), categorized by polymer type: polypropylene (PP), polyethylene (PE), polystyrene (PS), polyamide (PA), cellulose acetate (CA), polyethylene terephthalate (PET), and polyacrylonitrile (PAN).

## 4 Discussion

### 4.1 Guano Biogeochemical Particulate Composition

To assess the potential input of carbon by penguin guano in the SO ecosystem, we analyzed the biogeochemical particulate composition of guano from two species of Antarctic penguin, the Chinstrap and Gentoo, from Deception and Livingston Islands. We found that guano from the two penguin species exhibited broadly similar levels of POC and PIC, TN, and BSi. Existing information relating to POC in penguin guano is relatively scarce, and no data regarding on PIC concentrations are currently available in penguin guano samples. Previous studies only reported total organic carbon (indicated as “TC” or “TOC”) values of an average of 21 ± 7% (dw) in Chinstrap samples from South Shetland Islands,^44^ of up to 30% (dw) in Adélie penguin guano in the Ross Sea region,^45^ and of 12% (dw) in emperor penguin guano in eastern Antarctica,^46^ which are in accordance with our findings (16.1–37.2% dw). The presence of PIC in both species may derive from minor prey items, such as invertebrate with a carbonate shell, including mollusk gastropod,^47–49^ and from lithogenic fragments (i.e., rocks, stones, or pebbles). The stones found in penguin guano are primarily associated with their digestive processes. Penguins ingest stone, known as gastroliths, which serve various functions, including aiding in digestion and potentially assisting in deep diving, or either ingest accidently through dietary intake.^47,50^ The negligible levels of BSi are related to the fact that penguins do not directly consume diatoms, despite a minor amount likely being unintentionally ingested while feeding in seawater, as well as through consumption of krill, which in turn ingest diatoms.^51^ The observed higher POC:N ratio in Gentoo guano (2.6–5.0) reveals a relatively more carbon-rich material compared to Chinstrap guano (1.2–4.2), suggesting a different biochemical composition of the prey.^52,53^ Different dietary strategies (i.e., whether they feed more on krill or fish/squid) can influence their POC:N ratio.^54,55^ Furthermore, Gentoo penguins forage in coastal waters (<200 m water depth), performing both pelagic and especially benthic dives in response to local oceanographic and seafloor features.^56,57^ However, Chinstrap forage near the shelf break (>200 m water depth) in the epipelagic/mid layer, likely due to the high availability of krill, where oceanographic conditions promote the formation of dense aggregations of prey and continual replenishment.^56,58^ Guano biogeochemical composition can provide information on the distinctive trophic patterns of the two species, despite occupying a shared ecological niche and their similar feeding habits.^49^ In this study, POC content in penguin guano contributed on average to 26% of the total guano dry mass, which is very similar to the maximum value observed in krill feces (29%). In the SO, krill feces play a crucial role in the carbon cycle, contributing over 70% of the POC vertical flux.^16,51,58–60^ Consequently, penguin guano, similar to krill feces, could be considered an important source of POC in the SO, potentially entering and sustaining the benthic domain. Given the large Pygoscelid penguin population and the huge quantities of guano produced, penguins may represent key elements to conduit carbon export in SO. This is in accordance with the study of Gambi et al.^61^ which found an accumulation of particulate organic matter up to 70 m depth, in correspondence of one of the largest Adélie penguin colony in the Ross Sea region.

### 4.2 Guano Microplastic Composition

We found MPs in 91% of our guano samples (10 out of 11), which is in agreement with a previous study that found MPs in 77% of King penguin (*Aptenodytes patagonicus*) guano samples at South Georgia Island.^23^ In contrast, previous reports found lower MPs in penguin guano, ranging between 20% and 29% in Adélie, Chinstrap, and Gentoo penguins across the Antarctic Peninsula.^22,24^ However, all these studies focus on MPs larger than 63 μm. Here, we report for the first time the identification of smaller MP particles (down to 25 μm), a size fraction unexplored in penguin guano. We found 69 MPs, representing 46% of the total MPs, ranging between 25 and 50 μm. This small size range is of particular interest since MPs <50 μm represents the most abundant size fraction observed in the Southern Ocean. In sea surface water samples from the Weddell Sea, Leistenschneider et al.^33^ found the smallest MPs size representing 42% (11 μm) and 25% (25–50 μm) of the total MPs abundance. Jones-Williams et al.^32^ also reported that particles and fibers smaller than 50 μm accounted for 95% of the total MP abundance in Antarctic snow. Our results suggest that previous investigations on MPs in penguin guano may have underestimated the number of MP particles since those measuring less than 63 μm were not considered in previous studies due to procedural limitations. Furthermore, particular attention should be given to the small size range of MPs within fecal matter as larger particles are likely to be retained in the digestive system and/or fragmented prior to excretion.^62^ MPs observed here may derive from direct ingestion or may have originated from the breakdown of larger plastic fragments, which represent the most commonly ingested type of MPs by seabirds.^25,63,64^ MPs detected in krill span from 2 to 193 μm.^65,66^ Therefore, penguins may be exposed to MPs indirectly through trophic transfer via krill consumption.

Although guano sampling was not initially designed for microplastic analysis and minor background contamination cannot be completely ruled out, the consistency of our results with previous studies supports their overall reliability. Here, we found that MP particles were more abundant in Chinstrap (75 MPs) than in Gentoo (27 MPs) penguin guano samples, in accordance with the results of Fragão et al.^24^ Different foraging depths between the two species may also partly drive the difference in ingestion, accumulation, and excretion of MPs (in addition to the difference in diet discussed above). This has been observed for other organisms such as two sympatric species of coastal dolphins with different feeding habits in depths, studied in the Atlantic Ocean.^67^ Chinstrap penguins, which are epipelagic feeders, may have a greater encounter rate of plastic than Gentoo penguins, which feed predominantly in the benthic domain,^25,55^ because low-density MPs are typically buoyant in the water column. This could explain why we only found PET polymers in Gentoo penguin guano, given the PET polymer density (1.38 g cm^-^^3^) is higher than that of the seawater and therefore it is more readily available in benthos. We found PP to be the polymer with the highest frequency of occurrence (34% in Chinstrap and 75% in Gentoo), followed by PE and PA (37% and 18% in Chinstrap penguin). This is in agreement with previous revision studies, which found PP to be the most common polymer detected in Antarctic seabirds’ matrices (such as pellets and stomach samples) and in *Pygoscelis* penguin species, among others.^68^ More broadly, PP has been noted as the most ingested polymer by seabirds worldwide.^25^ Typically, PP is used for packaging, laboratory equipment, bottle caps, and the fishery industry.^69,70^ PA, the second most common polymer, is linked to textiles derived from technical clothing and equipment like ropes or route-marking flags.^32^ Its ubiquity in Antarctica is well-documented, for example, it is found in feces from three sympatric seal species of the Western Antarctic Peninsula,^71^ in penguins’ gastrointestinal tracks,^63^ and in snow from sites near the Antarctic research camp,^32^ indicating a local pollution source (such as the wastewater treatment plants of scientific stations).^72^ PE was found the most representative for Spheniscidae,^68^ in *Pygoscelis* guano samples and in the digestive tracks of Gentoo penguins’ chicks.^24,64^ It could be associated with ropes and gear of the fishery industry,^69^ which is among the main sources of plastic pollution in the Antarctic region. While the presence of MPs detected here was considerably higher than previously reported (such as number of items), their overall contribution in terms of mass can be similar since the dimensions cover a size range notably lower than those found in penguin guano elsewhere. For this reason, reporting both the total particles number and their corresponding mass is essential to enable proper comparison among studies as previous research on penguin guano reported MP abundance only, not providing the corresponding MP mass.

### 4.3 Biogeochemical Cycles Implication

Although the biogeochemical and MP fractions of guano were analyzed independently, their interaction has important implications for carbon cycling. The high particulate organic carbon (POC) and carbonate (PIC) contents indicate that penguin guano represents a dense, carbon-rich material with the potential to contribute to vertical carbon export. However, the coexistence of abundant small, low-density MP particles (e.g., PP and PE) within this organic matrix may alter the physicochemical properties of guano, affecting its compactness and sinking behavior (e.g., by increasing buoyancy). In this sense, MPs do not modify the chemical composition of guano directly but may influence its ballasting capacity and remineralization rate, ultimately modulating the efficiency of fecal matter-mediated carbon fluxes in the Southern Ocean.

Here, we show that penguin guano can represent a major source of carbon in coastal areas near the colonies. Therefore, a deeper understanding of the fate and vertical dynamics in the water column (sinking versus floating) of this rich-carbon fecal material is essential to assess the role of penguin guano in mediating carbon export from the ocean surface to the seafloor. The pulpy consistency of penguin guano is more similar to liquid whale feces than zooplankton fecal pellets, which often contain a peritrophic membrane, preventing the release of nutrients and ensuring a more efficient sinking rate.^73^ However, despite guano consistency (which will promote the recycling of organic matter in the surface ocean), part of guano will eventually start to sink (e.g., due to ballasting mechanisms for the presence of stones and fragments of organic matter such as krill exoskeletons), acting as a vector of carbon export to the deep ocean. Qualitative observation of guano content confirms the high presence of krill exoskeletons (Supporting Information, Figure S1), which are already considered an important vector for carbon fluxes in the Southern Ocean.^60^ Similarly, small guano particles can repack and aggregate with other particles, generating new formations, similar to marine snow, which consists of organic material and phytodetritus that incorporates with inorganic matter, resulting in higher sinking velocity.^74,75^ On the other hand, the high presence of small-size MPs found in this study potentially contributes to altering the stability, integrity, and compactness of penguin guano. Laboratory experiments have shown that the incorporation of low-density plastic fragments can alter the structure and density of organic matter, resulting in changes to sinking and remineralization rates, altering POC aggregation,^76,77^ thereby decreasing the carbon export to the deep sea. Given the amorphous nature of guano compared with structured fecal pellets, empirical sinking experiments will be required to confirm these physical interactions.

Based on an average guano production rate of 84.5 g of dry guano per individual per day^78^ and a POC content of 26% (this study), each penguin contributes approximately 21.97 g of POC daily through guano deposition. Over a 120 day breeding season, this corresponds to an individual export of ∼2.6 kg of POC, highlighting the potential role of penguin colonies in carbon fluxes. As for our results, MP particles represent an average of 0.3% of the total weight of POC plus MPs in the penguins’ guano. Thus, we can estimate daily export of MPs per individual to be ∼0.07 g. Over a 120 day breeding period, this corresponds to ∼8 g per penguin. However, upon scaling up to guano production across Chinstrap and Gentoo penguin populations in the South Shetland Islands (SSI) (about 986,440 Chinstrap individuals;^79^ 108,788 Gentoo individuals estimated from Herman et al.^80^), this small amount could be relatively important. Considering that approximately 54% of the total amount of guano produced may contain detectable MPs (data derived from reports on MPs and penguin guano, including the present study), the SSI population could contribute an estimate of 4.7 tons of MPs discharged for the breeding season. Once released into the seawater, MPs in the guano can re-enter biogeochemical cycles and become bioavailable again to zooplankton or undergo flocculation processes. In this context, penguins may act as a vector or transport mechanism for MPs injection in the water column. Moreover, plastic production is increasing globally;^81^ therefore, MPs in the SO are expected to rise, in particular in the northern region of the Antarctic peninsula, which is considered as a hotspot of ecological impact for MP accumulation.^26^ Consequently, as middle/top epipelagic predators, Antarctic penguins will likely ingest and excrete higher MPs projecting into the future. Furthermore, it is possible to calculate the MPs:POC ratio to evaluate the “natural” versus “anthropogenic” carbon (Table 1). Using an average carbon content of plastic of 82.68% (plastic carbon = total plastic × 0.8268),^82^ we calculated the mass ratio between plastic-derived carbon and natural POC (ratio = plastic carbon:POC). The MPs:POC ratio is 0.003 for Chinstrap and 0.001 for Gentoo, indicating that, although present, plastic carbon currently represents less than 0.4% of the total organic carbon pool in the guano. Therefore, while the ‘anthropogenic’ inclusion of carbon is detectable, it does not yet significantly alter the bulk density or the primary stoichiometry of the carbon export mediated by individuals.

**Table 1:** Estimation of the “Natural” versus “Anthropogenic” Carbon^a^ ^b^

Penguin Species	Total MP in Guano (mg)	Carbon Content of Plastic	Plastic Carbon (mg)	POC Guano (mg)	MPs:POC Ratio
Chinstrap	0.004	0.8268	0.003	0.96	0.003
Gentoo	0.0015	0.8268	0.001	0.99	0.001

a Calculation for the MPs:POC ratio in guano from Chinstrap and Gentoo penguins from Deception and Livingston Islands.

b Note: the carbon content of plastic was estimated in the study by Zhu et al.;^82^ the total MP in guano (mg) considered only the smallest fraction of MPs found in this study (not fibers).

Overall, this study helps boost the information on Chinstrap and Gentoo penguin guano composition in terms of biogeochemical and anthropogenic particular matter, providing new insight into the presence of the smallest class sizes of MP polymers in penguin guano, which had not been detected previously. Our findings suggest that penguin colonies may act as localized hotspots for organic carbon transfer at the terrestrial–maritime interface. Future research should quantify the proportion of guano that remains afloat versus the fraction that sinks, in order to distinguish between material recycled in surface waters and material contributing to carbon export to the deep ocean. On the other hand, our results highlight that penguins’ guano is acting as a vector of MPs in the SO ecosystem. The distinct trophic patterns of the two penguin species define the difference in the biochemical composition of their guano, for both carbon and MPs, thereby influencing its fate and the potential contribution to carbon export to the deep ocean. Identifying the main mechanisms that drive these pathways is essential to estimate the amount of carbon export mediated by guano and to understand how MPs pollution can interfere with the carbon sinking pathway.

## Supplementary Material



## Data Availability

The Supporting Information set is provided in the Supporting Information section and all data is available at NERC EDS UK Polar Data Centre (10.5285/88c79a01-d3c0-4fca-835c-481930948a5e).

## Notes

The supporting data set includes polymers identifiable by the Purency Microplastics Finder (MPF), polymer densities, and detailed information on microplastic polymers and particle masses (PDF)
