# Supplementary material for: From Carbon to Plastics: Partitioning of Biogeochemical and Anthropogenic Particles in Penguin Guano
Source: Environ Sci Technol. 2026 Jun 12;60(25):18070–9. doi: 10.1021/acs.est.5c17586 (PMC13325852; doi:10.1021/acs.est.5c17586)

Supporting Information for

From Carbon to Plastics: Partitioning of Biogeochemical and Anthropogenic Particles in Penguin Guano

Erica Sparaventi[1,2,3*], Emily Rowlands[1], Federico Giglio[4], Araceli Rodríguez-Romero[3], Antonio Tovar-Sánchez[3], Clara Manno[1*]

[1] British Antarctic Survey, High Cross, Madingley Rd, Cambridge CB3 0ET, United Kingdom of Great Britain and Northern Ireland

[2] Department of Genetic Toxicology and Cancer Biology, National Institute of Biology, Večna pot 121, 1000 Ljubljana, Slovenia

[3] Department of Ecology and Coastal Management, Institute of Marine Sciences of Andalusia, ICMAN (CSIC), Campus Río San Pedro, 11510, Puerto Real, Cádiz, Spain

[4] CNR-ISP – National Research Council of Italy – Institute of Polar Sciences, 40129 Bologna, Italy

Summary of Supplementary Information:

Total number of pages: 9

Total number of figures: 1

Total number of tables: 4

Contents:

Table S1. List of the 21 supported polymer types, identifiable by the Purency Microplastics Finder (MPF) (Purency GmbH, Austria).

Table S2. Polymer density information used for each polymer to calculate the mass of each MP particle found in penguin guano samples.

Table S3. Information on microplastic polymers found in Chinstrap and Gentoo penguin guano samples from Deception and Livingston Islands, and in the procedural blanks (PB), air contamination blanks (AIR) and the storage blanks (SB) derived by the Purency Microplastics Finder (MPF) (Purency GmbH, Austria). Sample items excluded from the results for blank corrections are marked in red. (Vapour Col: VC; Sally Rock: SR; Argentina Cove: AC; Hannah Point: HP).

Table S4. The small MP particles mass found in guano samples from Chinstrap and Gentoo penguins, and the % of MPs to the Total mass particulate (POC plus MPs), in µg.

Figures S1. Chinstrap penguin guano sample collected in Deception Island. The picture highlights the presence of feathers (yellow arrow) and parts of krill exoskeleton (orange arrow).

Table S1. List of the 21 supported polymer types, identifiable by the Purency Microplastics Finder (MPF) (Purency GmbH, Austria).

Systematic name	Class ID
Polypropylene	PP
Polyethylene	PE
Polyvinyl chloride	PVC
Polyurethane	PU
Polyethylene terephthalate	PET
Polystyrene	PS
Acrylonitrile butadiene styrene	ABS
Polyamide	PA
Polycarbonate	PC
Poly (methyl methacrylate)	PMMA
Polyoxymethylene	CA
Cellulose acetate	EVAc
Ethylene vinyl acetate	EVOH
Ethylene vinyl alcohol	PAN
Polyacrylonitrile	PBT
Polybutylene terephthalate	PEEK
Polyether ether ketone	POM
Polyphenylene sulfone	PPSU
Polysulfone	PSU
Silicone	Silicone
Polylactic acid	PLA

Table S2. Polymer density information used for each polymer to calculate the mass of each MP particle found in penguin guano samples.

Class ID	Polymer	Density (g cm^{-3})
PP	Polypropylene	0.95
PE	Polyethylene	0.95
PS	Polystyrene	1.03
PA	Polyamide	1.1

CA	Cellulose artificial modified	1.3
PAN	Polyacrylonitrile	1.18
PET	Polyethylene Terephtalate	1.38

Table S3. Information on microplastic polymers found in Chinstrap and Gentoo penguin guano samples from Deception and Livingston Islands, and in the procedural blanks (PB), air contamination blanks (AIR) and the storage blanks (SB) derived by the Purency Microplastics Finder (MPF) (Purency GmbH, Austria). Sample items excluded from the results for blank corrections are marked in red. (Vapour Col: VC; Sally Rock: SR; Argentina Cove: AC; Hannah Point: HP).

Sample ID	Penguin Species	Year	Colony	Class ID	Length (µm)	Width (µm)	Area (µm^2)	Relevance	Size (px)	volume (µm^3)
D1.3-G	Chinstrap	2021	VC	PP	74	29.81	1512.50	0.65	50	23613.57
D1.3-G	Chinstrap	2021	VC	PP	61	39.8	1603.25	0.66	53	20800.87
D1.3-G	Chinstrap	2021	VC	PE	49	38.93	1210.00	0.71	40	43538.58
D1.3-G	Chinstrap	2021	VC	PE	67	40.21	1512.50	0.71	50	128569.16
D1.3-G	Chinstrap	2021	VC	PE	58	46.06	1784.75	0.76	59	9315.77
D1.3-G	Chinstrap	2021	VC	PE	164	72.97	6897.00	0.77	228	81860.67
D1.3-G	Chinstrap	2021	VC	PE	41	29.22	726.00	0.64	24	11343.8
D1.3-G	Chinstrap	2021	VC	PE	127	70.05	4325.75	0.77	143	669.4
D1.3-G	Chinstrap	2021	VC	PE	39	28.41	786.50	0.76	26	8812.24
D1.3-G	Chinstrap	2021	VC	PE	51	13.11	363.00	0.66	12	61787.09
D1.3-G	Chinstrap	2021	VC	PE	44	27.97	786.50	0.66	26	75274.61
D1.3-G	Chinstrap	2021	VC	PE	71	57.28	2329.25	0.76	77	7862.49
D1.3-G	Chinstrap	2021	VC	PE	114	58.67	4083.75	0.7	135	12263.44
D1.3-G	Chinstrap	2021	VC	PS	141	71.12	6261.75	0.87	207	5417.41
D1.3-G	Chinstrap	2021	VC	PA	35	30.75	726.00	0.78	24	27860.81
D1.3-G	Chinstrap	2021	VC	PA	61	33.67	1179.75	0.62	39	5151.02
D1.3-G	Chinstrap	2021	VC	PA	30	22.89	423.50	0.63	14	10879.87
D1.3-G	Chinstrap	2021	VC	CA	33	25.18	484.00	0.73	16	16822.24
D1.3-G_2	Chinstrap	2021	VC	PP	242	142.63	22657.25	0.72	749	10474.69
D1.3-G_2	Chinstrap	2021	VC	PP	265	110.37	13521.75	0.87	447	772781.04
D1.3-G_2	Chinstrap	2021	VC	PP	199	134.91	18240.75	0.8	603	8880.82
D1.3-G_2	Chinstrap	2021	VC	PP	36	25.33	544.50	0.67	18	6475.79
D1.3-G_2	Chinstrap	2021	VC	PE	45	30.1	726.00	0.66	24	6830.63
D1.3-G_2	Chinstrap	2021	VC	PE	40	32.07	786.50	0.75	26	12616.58
D1.3-G_2	Chinstrap	2021	VC	PE	33	22.88	514.25	0.74	17	27278.17
D1.3-G_2	Chinstrap	2021	VC	PE	46	41.04	1210.00	0.72	40	1249004.33

Sample ID	Penguin Species	Year	Colony	Class ID	Length (μm)	Width (μm)	Area (μm²)	Relevance	Size (px)	volume (μm³)
D1.3-G_2	Chinstrap	2021	VC	PE	28	22.71	423.50	0.68	14	287663.06
D1.3-G_2	Chinstrap	2021	VC	PE	57	30.09	1089.00	0.72	36	1136015.23
D1.3-G_2	Chinstrap	2021	VC	PE	50	33.29	1119.25	0.75	37	5807.53
D1.3-G_2	Chinstrap	2021	VC	PE	30	28.14	574.75	0.7	19	13147.91
D1.3-G_2	Chinstrap	2021	VC	PE	177	137.57	13249.50	0.73	438	12702.04
D1.3-G_2	Chinstrap	2021	VC	PE	35	29.79	605.00	0.67	20	4534.28
D1.3-G_2	Chinstrap	2021	VC	PE	40	26.14	635.25	0.65	21	140104.57
D1.3-G_2	Chinstrap	2021	VC	PE	44	27.63	695.75	0.66	23	5563.88
D1.6-A	Chinstrap	2021	VC	PP	80	36.49	1542.75	0.78	51	13468.95
D1.6-A	Chinstrap	2021	VC	PP	144	93.79	6534.00	0.63	216	192381.25
D1.6-A	Chinstrap	2021	VC	PP	44	32.57	877.25	0.61	29	12783.49
D1.6-A	Chinstrap	2021	VC	PP	77	33.17	1421.75	0.62	47	11267.63
D1.6-A	Chinstrap	2021	VC	PP	61	36.36	1119.25	0.73	37	12333.88
D1.6-A	Chinstrap	2021	VC	PP	41	24.33	635.25	0.67	21	5921.28
D1.6-A	Chinstrap	2021	VC	PP	83	75.99	3509.00	0.63	116	135396.56
D1.6-A	Chinstrap	2021	VC	PP	29	17.4	332.75	0.68	11	2325.97
D1.6-A	Chinstrap	2021	VC	PP	52	40.69	1240.25	0.67	41	23102.8
D1.6-A	Chinstrap	2021	VC	PP	91	48.57	3206.50	0.78	106	60476.9
D1.6-A	Chinstrap	2021	VC	PP	100	36.04	1845.25	0.61	61	12264.07
D1.6-A	Chinstrap	2021	VC	PP	42	25.53	605.00	0.62	20	5417.98
D1.6-A	Chinstrap	2021	VC	PP	126	69.51	5535.75	0.61	183	133344.41
D1.6-A	Chinstrap	2021	VC	PE	79	45.78	2026.75	0.71	67	40901.2
D1.6-A	Chinstrap	2021	VC	PE	30	25.29	453.75	0.71	15	187925.77
D1.6-A	Chinstrap	2021	VC	PE	50	37.69	1452.00	0.68	48	20691.65
D1.6-A	Chinstrap	2021	VC	PE	70	30.07	1240.25	0.74	41	32922.6
D1.6-A	Chinstrap	2021	VC	PE	319	106.89	20146.50	0.76	666	28492.28
D1.6-A	Chinstrap	2021	VC	PS	88	42.1	2299.00	0.63	76	72157.14
D1.6-A	Chinstrap	2021	VC	PA	49	31.8	574.75	0.61	19	139626.4
D1.6-A	Chinstrap	2021	VC	PA	33	19.53	393.25	0.65	13	4246.35
D1.6-A	Chinstrap	2021	VC	PA	35	25.28	393.25	0.61	13	5217.52
D1.6-A	Chinstrap	2021	VC	PA	46	13.79	363.00	0.63	12	10542.22
D1.6-A	Chinstrap	2021	VC	PA	50	16.57	453.75	0.62	15	4420.14
D1.6-A	Chinstrap	2021	VC	PA	42	22.86	665.50	0.63	22	2775.08
D1.6-A	Chinstrap	2021	VC	PA	39	13.08	332.75	0.62	11	3218.91
D1.6-A	Chinstrap	2021	VC	PA	92	57.12	2117.50	0.62	70	876.18
D1.6-A	Chinstrap	2021	VC	PA	37	17.72	393.25	0.64	13	1388.97
D1.6-A	Chinstrap	2021	VC	PA	29	21.75	393.25	0.65	13	5830.76
D1.6-A	Chinstrap	2021	VC	PA	536	268.34	57444.75	0.63	1899	955.6
D1.6-A	Chinstrap	2021	VC	PA	44	27.72	605	0.6	20	29965.48
D1.6-A	Chinstrap	2021	VC	PA	61	34.25	847.00	0.65	28	2026.81
D1.6-A	Chinstrap	2021	VC	PA	37	19.54	393.25	0.62	13	3867.65

Sample ID	Penguin Species	Year	Colony	Class ID	Length (µm)	Width (µm)	Area (µm²)	Relevance	Size (px)	volume (µm³)
D1.6-A	Chinstrap	2021	VC	PA	50	33.14	816.75	0.6	27	3082169.54
D1.6-A	Chinstrap	2021	VC	PA	35	17.77	363.00	0.66	12	5217.07
D1.6-A	Chinstrap	2021	VC	PA	44	22.06	635.25	0.6	21	6655.67
D1.6-A	Chinstrap	2021	VC	PA	39	29.96	605.00	0.61	20	2215.66
D1.6-A	Chinstrap	2021	VC	PA	44	20.85	393.25	0.65	13	8942.7
D1.6-A	Chinstrap	2021	VC	PA	44	22.16	514.25	0.62	17	1899.5
D1.6-A	Chinstrap	2021	VC	PA	33	27.5	484.00	0.63	16	4585.7
D1.6-A	Chinstrap	2021	VC	PA	84	17.86	907.50	0.6	30	7239.47
D1.6-A	Chinstrap	2021	VC	PA	45	15.23	393.25	0.63	13	1642.99
D1.6-A	Chinstrap	2021	VC	PA	506	329.26	64160.25	0.61	2121	3029.76
D1.6-A	Chinstrap	2021	VC	PA	35	15.56	332.75	0.61	11	5915.56
D1.6-A_2	Chinstrap	2021	VC	PP	80	48.14	2329.25	0.68	77	2073.69
D1.6-A_2	Chinstrap	2021	VC	PP	111	87.02	5142.50	0.85	170	1165.68
D1.6-A_2	Chinstrap	2021	VC	PP	45	33.97	1119.25	0.73	37	5296342.34
D1.6-A_2	Chinstrap	2021	VC	PP	77	49.2	1996.50	0.66	66	1405.6
D1.6-A_2	Chinstrap	2021	VC	PP	72	44.39	1815.00	0.82	60	6061.29
D1.6-A_2	Chinstrap	2021	VC	PP	113	67.53	3690.50	0.65	122	5025.62
D1.6-A_2	Chinstrap	2021	VC	PP	116	65.93	5324.00	0.66	176	4142.84
D1.6-A_2	Chinstrap	2021	VC	PP	25	20.66	363.00	0.7	12	21336.41
D1.6-A_2	Chinstrap	2021	VC	PP	28	22	423.50	0.76	14	6485.37
D1.6-A_2	Chinstrap	2021	VC	PP	82	34.94	1421.75	0.7	47	133159.95
D1.6-A_2	Chinstrap	2021	VC	PE	72	52.25	2692.25	0.8	89	2407.23
D1.6-A_2	Chinstrap	2021	VC	PE	48	37.57	1270.50	0.79	42	55245.5
D1.6-A_2	Chinstrap	2021	VC	PE	35	17.64	393.25	0.61	13	7248.28
D1.6-A_2	Chinstrap	2021	VC	PE	44	15.63	453.75	0.77	15	5569.12
D1.6-A_2	Chinstrap	2021	VC	PS	40	32.28	847.00	0.61	28	6600.29
D1.6-A_2	Chinstrap	2021	VC	PS	165	92.4	8591.00	0.83	284	11458.12
D1.6-A_2	Chinstrap	2021	VC	PA	33	24.96	514.25	0.64	17	26725.17
D1.6-A_2	Chinstrap	2021	VC	PA	38	22.41	574.75	0.62	19	35810.3
D1.6-A_2	Chinstrap	2021	VC	PA	38	22.86	514.25	0.66	17	8874.26
D1.6-A_2	Chinstrap	2021	VC	PA	57	37.7	1361.25	0.69	45	2872.04
D1.6-A_2	Chinstrap	2021	VC	PA	30	24.47	484.00	0.63	16	73116.28
D1.6-A_2	Chinstrap	2021	VC	PA	80	69.49	3509.00	0.76	116	26442.4
D1.6-A_2	Chinstrap	2021	VC	PA	37	25.24	363.00	0.62	12	2223.08
D1.6-A_2	Chinstrap	2021	VC	PA	83	65.63	2420.00	0.64	80	1633.13
D1.6-A_2	Chinstrap	2021	VC	PA	60	30.09	937.75	0.64	31	30439.05
D1.6-A_2	Chinstrap	2021	VC	PA	29	25.31	423.50	0.62	14	5766.25
D1.6-A_2	Chinstrap	2021	VC	PA	47	27.1	726.00	0.63	24	31989.19
D1.6-A_2	Chinstrap	2021	VC	PA	29	28.83	574.75	0.61	19	9356.59
D1.6-A_2	Chinstrap	2021	VC	PA	50	38.32	1331.00	0.66	44	426793.64
D1.6-A_2	Chinstrap	2021	VC	PA	94	52.76	2450.25	0.6	81	28851.82

Sample ID	Penguin Species	Year	Colony	Class ID	Length (µm)	Width (µm)	Area (µm²)	Relevance	Size (px)	volume (µm³)
D1.6-A_2	Chinstrap	2021	VC	PA	30	29.8	514.25	0.61	17	14372.94
D1.6-A_2	Chinstrap	2021	VC	PA	31	20.69	363.00	0.62	12	250490.65
SD1.6	Chinstrap	2021	VC	PAN	>5000	197.05		0.6		152479958
SD1.6	Chinstrap	2021	VC	PAN	>5000	140.81		0.6		77866977.8
D2.4-C	Chinstrap	2021	VC	PP	30	19.36	393.25	0.72	13	3337.71
D2.4-C	Chinstrap	2021	VC	PP	33	25.21	574.75	0.86	19	7665.78
D2.4-C	Chinstrap	2021	VC	PP	79	16.65	635.25	0.77	21	1083.43
D2.4-C	Chinstrap	2021	VC	PP	39	34.55	907.50	0.79	30	18755.4
D2.4-C	Chinstrap	2021	VC	PP	33	25.25	453.75	0.71	15	4779.62
D2.4-C	Chinstrap	2021	VC	PP	409	226.57	47159.75	0.89	1559	3010684.19
D2.4-C_2	Chinstrap	2021	VC	PP	33	24.75	574.75	0.73	19	7567.17
D2.4-C_2	Chinstrap	2021	VC	PP	106	91.27	7290.25	0.91	241	435160.61
D2.4-C_2	Chinstrap	2021	VC	PP	41	21.74	574.75	0.72	19	4333.27
D2.4-C_2	Chinstrap	2021	VC	PP	47	37.14	1179.75	0.78	39	23301.29
D2.4-C_2	Chinstrap	2021	VC	PP	54	40.28	1331.00	0.87	44	24362.96
SD2.4	Chinstrap	2021	VC	PA	2000	37.4		0.600		2197167.07
D2.5-D	Chinstrap	2021	VC	PE	37	22.765	484.00	0.694	16	3794.87
D2.5-D	Chinstrap	2021	VC	PS	83	48.259	2722.50	0.776	90	51724.41
D2.5-D_2	Chinstrap	2021	VC	PP	72	50.956	2571.25	0.669	85	65486.98
SD2.5D	Chinstrap	2021	VC	PET	1500	50.8947		0.6		3051594.94
D3.2-F	Chinstrap	2021	VC	PP	31	25.25	574.75	0.62	19	8902.1
D3.2-F	Chinstrap	2021	VC	PS	63	49.07	1694.00	0.64	56	146102.7
D3.2-F_2	Chinstrap	2021	VC	PP	113	66.89	5293.75	0.72	175	166157.74
D3.2-F_2	Chinstrap	2021	VC	PP	313	83.92	13915.00	0.79	460	235980.29
D3.2-F_2	Chinstrap	2021	VC	PP	114	102.48	5475.25	0.85	181	35098.35
D3.2-F_2	Chinstrap	2021	VC	PS	92	52.37	2813.25	0.68	93	49408.66
D3.2-F_2	Chinstrap	2021	VC	PS	44	36.84	1179.75	0.67	39	26044.78
SR.1	Gentoo	2022	SR	PP	139	42.292	3660.25	0.777	121	29219.35
SR.1	Gentoo	2022	SR	PS	50	37.538	1300.75	0.859	43	494658.75
SR.1	Gentoo	2022	SR	PS	54	34.564	1058.75	0.682	35	3969.11
SR.1_2	Gentoo	2022	SR	PP	145	107.9	9831.25	0.81	325	25578.61
SR.1_2	Gentoo	2022	SR	PP	33	24.54	423.50	0.67	14	13095.58
13.2	Gentoo	2022	AC	PP	57	50.32	1936.00	0.67	64	57423.61
13.2	Gentoo	2022	AC	PS	57	52.76	1936.00	0.68	64	5518442.95
13.2_2	Gentoo	2022	AC	PP	500	282.15	69907.75	0.77	2311	17106.32
13.2_2	Gentoo	2022	AC	PP	47	33.51	1058.75	0.71	35	5975.21
13.2_2	Gentoo	2022	AC	PP	30	22.88	484.00	0.74	16	99362.64
13.2_2	Gentoo	2022	AC	PP	61	60.11	2480.50	0.72	82	5306.47
13.2_2	Gentoo	2022	AC	PP	27	22.31	423.50	0.66	14	60672.99
13.2_2	Gentoo	2022	AC	PET	53	21.22	756.25	0.7	25	4389.7
27.2	Gentoo	2022	AC	PP	37	19.72	514.25	0.62	17	3788.85

Sample ID	Penguin Species	Year	Colony	Class ID	Length (μm)	Width (μm)	Area (μm²)	Relevance	Size (px)	volume (μm³)
27.2	Gentoo	2022	AC	PP	52	29.07	1058.75	0.74	35	11936.01
27.2	Gentoo	2022	AC	PA	28	16.5	332.75	0.7	11	11721.33
27.2_2	Gentoo	2022	AC	PP	36	32.03	695.75	0.86	23	2415.76
27.2_2	Gentoo	2022	AC	PET	62	33.12	1300.75	0.64	43	14625.03
25.2	Gentoo	2022	HP	/	/	/	/	/	/	/
25.2_2	Gentoo	2022	HP	/	/	/	/	/	/	/
1.3	Gentoo	2022	AC	PP	78	33.43	1119.25	0.68	37	6924.15
1.3	Gentoo	2022	AC	PP	87	66.99	4114.00	0.82	136	150869.19
1.3	Gentoo	2022	AC	PP	56.2	37.09	1482.25	0.77	49	25846.41
1.3_2	Gentoo	2022	AC	PP	170	142.46	14217.50	0.81	470	995129.78
1.3_2	Gentoo	2022	AC	PP	45	34.33	998.25	0.78	33	17037.37
S1.3	Gentoo	2022	AC	PET	>5000	57.77		0.6		23588368.4
15.2	Gentoo	2022	AC	PP	58	18.05	635.25	0.69	21	2190.11
15.2	Gentoo	2022	AC	PP	121	72.24	5717.25	0.77	189	160219.26
15.2_2	Gentoo	2022	AC	PP	52	39.94	1542.75	0.69	51	34846.5
15.2_2	Gentoo	2022	AC	PP	141	75.02	6715.50	0.76	222	169381.14
S15.2	Gentoo	2022	AC	PET	1300	27.51		0.6		772943.17
PB1				PA	40.6	33.0	756.25	0.64	25	
PB1				PA	37.4	25.3	544.50	0.70	18	
PB2				PA	51.9	44.3	1603.25	0.62	53	
PB3				PA	150.8	87.3	8863.25	0.68	54	
PB3				PA	32.6	22.7	453.75	0.67	293	
PB3				PE	53.7	46.2	1633.50	0.84	15	
AIR1				PE	49.8	40.2	1210.00	0.74	40	
AIR1				PA	77.2	45.1	2147.75	0.71	71	
AIR1				PA	40.2	24.3	605.00	0.67	20	
AIR1				PA	30.1	27.6	605.00	0.71	20	
AIR1				PA	37.1	22.9	423.50	0.62	14	
AIR2				PA	115.0	57.3	3569.50	0.72	118	
AIR2				PA	82.0	45.3	1875.50	0.62	62	
SB1				PA	39.9	20.8	544.50	0.73	18	
SB1				PA	62.6	45.4	1663.75	0.61	55	

Table S4. The small MP particles mass found in guano samples from Chinstrap and Gentoo penguins, and the % of MPs to the Total mass particulate (POC plus MPs), in µg

Species	PP	Mass PP (µg)	PE	Mass PE (µg)	PS	Mass PS (µg)	PA	Mass PA (µg)	CA	Mass CA (µg)	PET	Mass PET (µg)	MP total mass (µg)	Guano total weight (mg)	MP/guano (µg mg^{-1})	POC (µg)	Total Mass Particulate (µg)	MP/TOT (%)
Chinstrap	6	2.6	22	1.3	1	0.1			1	0.01			4.0	1003.8	0.004	972.3	976.3	0.4
Chinstrap	23	1.1	8	0.5	3	0.3	31	9.67					11.6	2642.0	0.004	747.7	759.3	1.5
Chinstrap	11	3.4											3.4	2680.2	0.001	1021.0	1024.4	0.3
Chinstrap	1	0.1	1	0.004	1	0.1							0.1	1523.4	0.0001	994.0	994.2	0.01
Chinstrap	4	0.5			3	0.1							0.6	1125.4	0.001	1043.7	1044.4	0.1
Gentoo	3	0.5			2	0.04							0.5	1204.2	0.0004	941.8	942.3	0.1
Gentoo	6	5.4			1	0.1					1	0.01	5.5	1528.1	0.004	889.8	895.3	0.6
Gentoo	3	0.03									1	0.02	0.05	897.5	0.0001	1070.6	1070.7	0.004
Gentoo														1065.5		687.5	687.5	
Gentoo	5	1.14											1.1	1039.5	0.001	1030.5	1031.6	0.1
Gentoo	4	0.35											0.3	1010.1	0.0003	1307.0	1307.3	0.03

Figure S1. Chinstrap penguin guano sample collected in Deception Island. The picture highlights the presence of feathers (yellow arrow) and parts of krill exoskeleton (orange arrow).

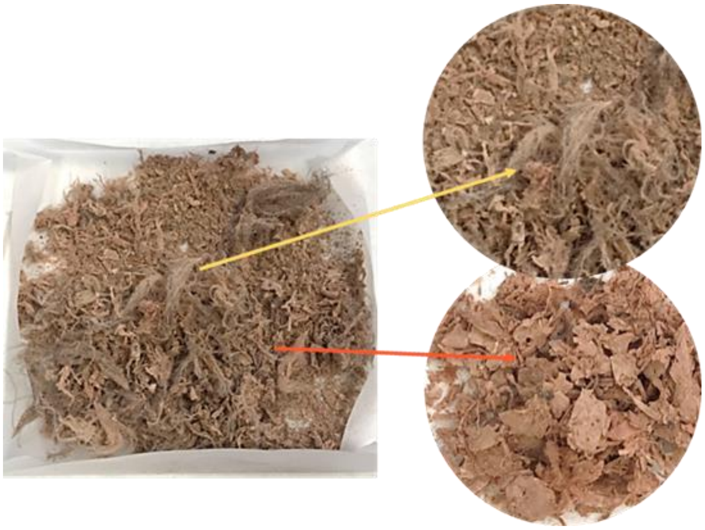

Supplement: Supplementary file 1 [file es5c17586_si_001.pdf]
